# Treatment Retention and Safety of Ixekizumab in Psoriatic Arthritis: A Real Life Single-Center Experience

**DOI:** 10.3390/jcm12020467

**Published:** 2023-01-06

**Authors:** Ignacio Braña, Estefanía Pardo, Stefanie Burger, Pablo González del Pozo, Mercedes Alperi, Rubén Queiro

**Affiliations:** 1 Rheumatology Division, Hospital Universitario Central de Asturias (HUCA), 33011 Oviedo, Spain; 2 ISPA Translational Immunology Division & Oviedo University School of Medicine, 33011 Oviedo, Spain

**Keywords:** psoriatic arthritis, ixekizumab, drug survival, safety, biologic therapy

## Abstract

Background and objectives: Information on the performance of ixekizumab (IXE) in patients with psoriatic arthritis (PsA) in clinical practice is scarce. We aimed to analyze the retention rate and safety of IXE in patients with PsA in routine clinical practice. Methods: A retrospective longitudinal observational single-center study of all patients with PsA who had received at least one dose of IXE. Adverse events (AEs) and drug retention rate were the main study focus. Survival was analyzed using Kaplan–Meier curves and predictive factors using multivariate Cox regression analysis. The hazard ratio (HR) was used as a measure of the association. Results: Seventy-two patients were included (52 women and 20 men). Median disease duration was 5 years (IQR 3–9). More than 90% received ≥2 biologic and/or targeted synthetic disease-modifying anti-rheumatic drugs (DMARDs) prior to IXE. Ixekizumab showed a 1-year retention rate of 65% and a 2-year retention rate of 57%. Regarding discontinuation due to AEs, 0.18 AEs per person-year were identified. The number of previous biologics did not influence drug survival but prior use of methotrexate (HR 2.31 (95% CI 1.05–5.10), *p* < 0.05) and depression (HR 2.40 (95% CI 1.07–5.41), *p* < 0.05) increased the risk of IXE discontinuation. Conclusions: Ixekizumab showed a good retention rate in a PsA population mostly refractory to biologic and targeted synthetic DMARDs. Drug survival was consistently good regardless of age, gender, metabolic comorbidities, smoking status, or prior number of biologic therapies. This information may be of interest to better position this drug in the PsA treatment algorithms.

## 1. Introduction

Psoriatic arthritis (PsA) is a chronic inflammatory arthritis that affects approximately one-third of psoriasis patients. Its varied and complex phenotypic expression is not only a diagnostic challenge for clinicians, but above all, a challenge when deciding the best treatment option for each patient. This is further complicated if we consider that the clinical phenotype at disease presentation can change over time, causing the initial treatment schemes to lose their original effectiveness [1].

Psoriatic arthritis management strategies are not homogeneous. The Group for Research and Assessment of Psoriasis and Psoriatic Arthritis (GRAPPA) has based its management recommendations on the most affected PsA domain [2], while the European Alliance of Associations for Rheumatology (EULAR) has opted for a more classic step-up scheme depending on whether the treatment objectives have been achieved or not [2,3]. Over the past decade, treatment options for PsA have expanded with the availability of many more novel therapeutic agents. However, the treatment decisions and pathways for the use of these drugs are not always straightforward [4].

The profile of patients entering randomized clinical trials (RCTs) is usually quite homogeneous from a clinical (polyarticular forms with elevated acute inflammatory markers) and therapeutic standpoint (patients naïve to biologics or previously exposed to only one or two of these therapies). However, the behavior of the new therapeutic molecules in the real clinical scenario (patients using several drugs, with comorbidities, with previous failures to three or more lines of biologic therapy, etc.) is basically unknown when these new targets reach the therapeutic market and are ready for use in clinical practice.

Ixekizumab (IXE) is an IgG4 therapeutic monoclonal antibody directed against interleukin-17A (IL17A) that has shown efficacy and safety in biologic-naïve PsA patients (SPIRIT P1) and in those previously exposed to anti-TNFα (SPIRIT P2), and it has shown superiority to adalimumab in a combined outcome PASI100 + ACR50 (SPIRIT H2H) [5,6,7]. Ixekizumab adequately covers all musculoskeletal and cutaneous domains of the disease, although its position within disease management schemes is not entirely clear. In general, it is usually relegated to the treatment of those cases where, apart from active arthritis, there is marked skin involvement [2,3]. To date, there are very few studies that have explored the benefits and limitations of this agent under real clinical conditions. Therefore, it is necessary to further explore how this new agent behaves in situations not contemplated in RCTs to obtain quality information that allows an adequate positioning of this drug in PsA. For example, it has recently been possible to show that the persistence rate of secukinumab (SEC), another anti-IL17A agent, is especially good in obese patients with PsA [8]. In addition, obese, hypertensive, and/or diabetic patients with PsA or axial spondyloarthritis have shown better SEC survival curves [9]. However, information in that sense is unknown with IXE.

The present study has analyzed drug survival and safety of IXE in patients with PsA under real clinical practice conditions. This information may complement that obtained from RCTs carried out to date with this IL17-blocking agent.

## 2. Patients and Methods

This was a single-center retrospective observational study. The project adhered to the postulates of the Declaration of Helsinki and the ethics review board of Hospital Universitario Central de Asturias and Principality of Asturias approved the study. Patients were informed about the objectives of the study but were exempted from written informed consent due to the retrospective nature of the study.

### 2.1. Study Population

All adult patients with PsA fulfilling the Classification Criteria for Psoriatic Arthritis (CASPAR) [10] who had received at least one dose of IXE from January 2019 to December 2021 were included. Ixekizumab prescription was made in accordance with the local regulatory laws of the health care system of the Principality of Asturias (northern Spain). In our setting, patients with PsA who require a biological therapy should start with a biosimilar anti-TNFα agent. If there is a contraindication for the use of these agents, or there is a lack of therapeutic response, a second line of treatment is opened, with a JAK inhibitor (tofacinitib) or IXE as potential options, with one or the other being chosen at the discretion of the prescribing physician. The use of IXE in this study was primarily as monotherapy, unless otherwise stated.

The main study outcomes were safety and drug survival. Safety was analyzed by reviewing the clinical charts from the start of IXE, as well as the hospital admission records; paying special attention to infections, neoplasms, and events located in or affecting any other organs or systems. Drug survival (in months) was defined as the time from the start of IXE to the last dose administered if discontinued or to the last dose administered if lost to follow-up. The reason and rate of suspension were also collected.

The following variables were collected: other treatments, disease features, comorbidities, form of disease onset, tobacco, age, years of disease evolution, sex, enthesitis, dactylitis, diabetes mellitus (DM), hypertension (HT), obesity, dyslipidemia (DL), depression, chronic obstructive pulmonary disease (COPD), major adverse cardiovascular events (MACEs), renal and hepatic insufficiency, biologic treatment line (1st line, 2nd, 3rd, 4th, etc.), previous adverse events (AEs), previous disease-modifying antirheumatic drugs (DMARDs) (yes/no and concomitant), and musculoskeletal as well as skin disease activity assessments (baseline and at 6 and 12 months).

### 2.2. Statistical Analysis

The sample was described in terms of the distribution of the descriptive variables by summary statistics. The rate of AEs was estimated in total, by severity and by type of event. The denominator used was the total number of patient-years of follow-up. Survival was analyzed using Kaplan–Meier curves and the hazard ratio (HR) was used as a measure of the association. Multivariate Cox regression was used to analyze the effect of the explanatory variables on drug survival. Potential confounding variables were age, disease duration, sex, previous treatments, and comorbidities. A study sample of 68 individuals was considered sufficient to estimate an expected AE rate of around 15% and a replacement rate of 5%, with 95% confidence and a precision of ±5 percentage units. Level of statistical significance was set at *p* < 0.05. Statistical analysis was carried out using the R statistical software.

## 3. Results

The study sample included 72 patients (52 women and 20 men). Mean age at psoriasis onset was 37.2 ± 13.1 years while mean age at arthritis debut was 44 ± 11.9 years. The distribution of joint patterns was as follows: axial in 11.1%, mixed in 27.8%, and peripheral in 61.1% of patients. Table 1 summarizes the main features of the study population.

In most patients (>90%), IXE was the 3rd or subsequent line of treatment. Among these 72 patients, IXE was stopped in 28 cases (38.9%). Of the patients who discontinued the drug, in 13 cases (46.4%) the cause was AEs, in 14 (50%) it was lack or loss of therapeutic response, and in one, it was preventively suspended due to positivity for cervical human papillomavirus. Regarding discontinuation due to AEs, 0.18 AEs per person-year were identified. That is, one adverse event for every 5.6 person-years of exposure. Most patients who discontinued due to AEs (75%) did so during the first 6 months of treatment. The most frequent AEs were gastrointestinal (nausea, diarrhea, abdominal pain), cutaneous (mainly injection site reactions and generalized rash), and infectious (mucocutaneous candidiasis, pneumonia, otitis, dental infection). Crohn’s disease was diagnosed in one patient during the exposure. Table 2 shows a description of the AEs identified, including those leading to drug discontinuation.

**Figure 1:**
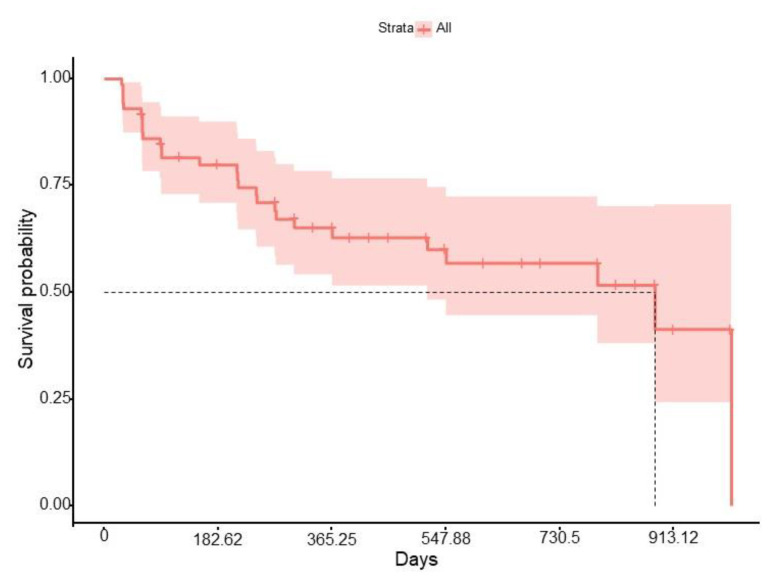
Ixekizumab survival curve.

**Figure 2:**
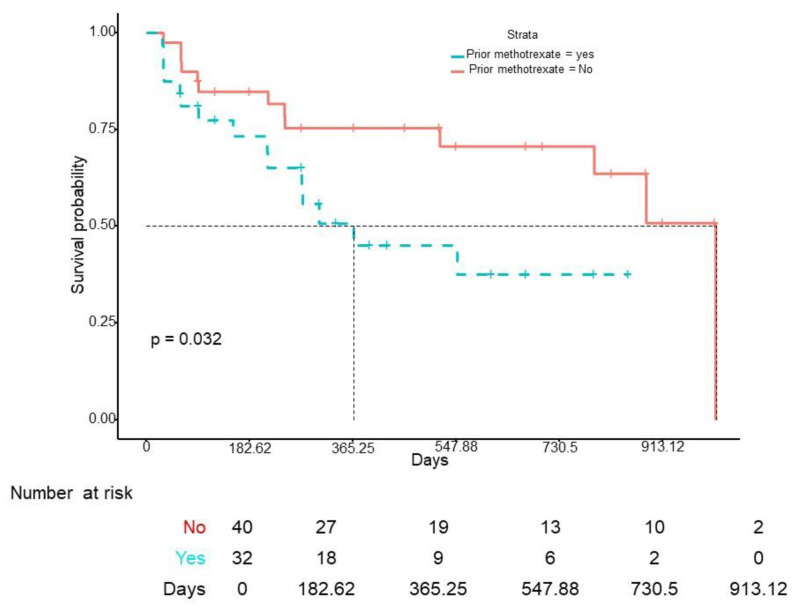
Ixekizumab survival curve according to previous exposure to methotrexate.

**Figure 3:**
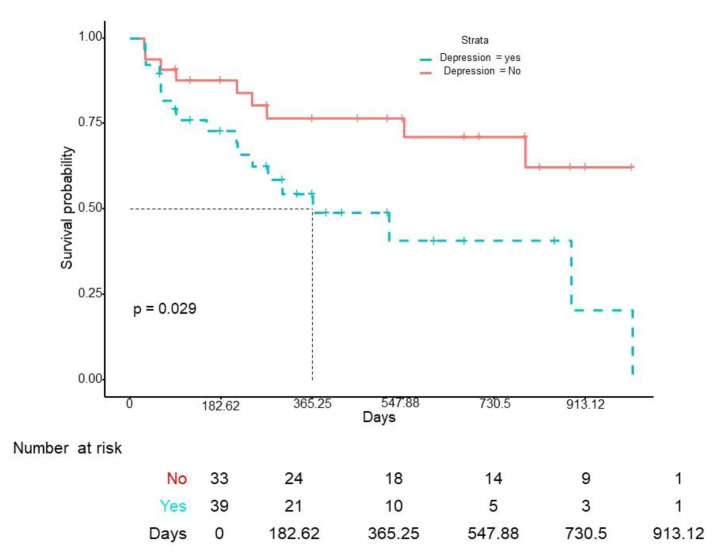
Ixekizumab survival curve according to the presence of depression.

The median survival of IXE was 9.2 months (IQR 3.07–18.8), with a 1-year retention rate of 65% and a 2-year retention rate of 57% (Figure 1). No differences in drug survival were found according to gender (log-rank *p* = 0.34), obesity (log-rank *p* = 0.24), smoking (log-rank *p* = 0.32), DM (log-rank *p* = 0.87), HT (log-rank *p* = 0.72), or DL (log-rank *p* = 0.14). However, previous use of methotrexate (HR 2.31 (95% CI 1.05–5.10), *p* < 0.05) and depression (HR 2.40 (95% CI 1.07–5.41), *p* < 0.05) were independently associated with a higher risk of IXE discontinuation (Figure 2 and Figure 3). When IXE survival was compared according to the line of biologic therapy, there were no differences in drug persistence when it was used as 2nd, 3rd, or subsequent lines of treatment (log-rank *p* = 0.39).

## 4. Discussion

In this single-center study, IXE demonstrated a high persistence rate at 1 (65%) and 2 years (57%) in a population of PsA previously exposed and mostly refractory to various lines of biologic and targeted synthetic drugs. Patients discontinued the drug in a similar proportion for both AEs and lack of effectiveness. Adverse events leading to discontinuation occurred mainly in the first 6 months of treatment. The number of previous biologics did not have any influence on drug survival while prior exposure to methotrexate was associated with higher risk of IXE discontinuation. The only comorbid factor associated with lower survival of IXE was depression, while none of the cardiometabolic risk factors, including obesity and smoking, affected the drug survival rate.

The sequential use of biologic therapies and switching between them is common in rheumatology practice. The lack or loss of therapeutic response is, unfortunately, a common phenomenon in the management of inflammatory arthritis, and PsA is not exempt from this consideration [11,12,13]. Therefore, it is essential in clinical practice to determine which factors are associated with greater or worse persistence of these agents, since many of these factors are not considered in most RCTs. Thus, among the main factors related to reduced TNFα inhibitor (TNFi) survival, different studies have found female sex, shorter disease duration, number of previous biologics, older age, current smoking, and comorbidities [11,12,13]. Female sex, number of previous biologics, and depression have also been related to poorer survival of SEC in real world evidence studies [9]. Patients who start IXE retain the drug in a large percentage for at least 12 months according to most RCTs carried out to date [5,6,7], but long-term data regarding IXE survival in daily clinical practice are limited [14,15]. Unlike other studies with biologics [9,11,12,13], we did not detect significant alterations of IXE survival rate in relation to age, gender, smoking, duration of illness, or metabolic comorbidities (including obesity).

A striking fact of our study was the predictive capacity of previous exposure to methotrexate in terms of lower persistence of IXE. These data are especially curious if we consider that the concomitant use of methotrexate tends to prolong the survival of TNFi in PsA [16]. The hypothesis put forward in these studies is that methotrexate would reduce the immunogenicity against TNFi, allowing a higher survival of these drugs [16]. However, clinical trials with IXE show that the associated use of methotrexate has no role in either the therapeutic response or the persistence of the drug [5,6,7]. In fact, our patients used IXE in monotherapy. In any case, we must be cautious when interpreting our methotrexate data as a predictor of worse persistence of IXE, since these data refer to prior methotrexate use both as monotherapy and in combination with TNFi.

Obesity may contribute to an increased risk of developing immune-mediated inflammatory diseases (IMIDs), and indeed, a considerable number of IMID patients are obese [17,18]. Moreover, a higher body mass index (BMI) is associated with accelerated TNFi clearance, resulting in lower trough concentrations [18]. On the other hand, obese patients with PsA are less likely to achieve treatment goals, more likely to discontinue treatment, and show lower skin clearance rate [17,18]. This effect appears to be proportional to BMI and, in fact, weight reduction increases the chances of response to treatment [18,19]. However, recently published studies with SEC, another IL17A inhibitor, show that neither obesity nor any other element of the metabolic syndrome (MetS) appears to negatively influence survival of this therapeutic class [8,9]. In fact, at least with SEC, just the opposite is true [8,9]. Our results with IXE seem to be along the same lines since none of the elements of MetS exert any influence on drug survival. Delving into the mechanisms of the positive interaction between this therapeutic class and MetS is beyond the objectives of this discussion, but our data and those of others underline the relevant role of IL17A in the metabolic inflammation (meta-inflammation) of PsA, and how this knowledge can be used to guide treatment decisions [20]. However, it remains to be shown whether this better persistence of IL17 inhibitors in patients with PsA and concomitant MetS extends to other members of this therapeutic class [21].

Our study identified depression as a risk factor for reduced drug survival among patients with PsA treated with IXE. These data are consistent with those of other studies showing that depression negatively affected the response to TNFi and was correlated with higher baseline disease activity and shorter TNFi persistence [22,23]. Psoriatic arthritis depressed patients treated with SEC also show worse drug survival [9]. Therefore, depression appears to be a universal predictor of poorer persistence for most biologics used in PsA.

This study has several limitations. First, this study comes from a single center where a specific protocol for the use of biologic and targeted synthetic DMARDs for PsA is applied, which means that its results cannot be generalized. On the other hand, it is a study with a retrospective collection of information, and with a relatively low number of patients, which surely limits the quality of the data retrieved. However, this study provides valuable add-on information of IXE’s benefits in patient profiles that are not usually considered in RCTs (comorbidities, polypharmacy, patients previously exposed to various biologic and targeted synthetic DMARDs, etc.).

## 5. Conclusions

Ixekizumab, an anti-IL17A therapy, demonstrated high drug persistence in a refractory PsA population. In addition, the aspects that usually penalize the survival of most biologics in PsA (age, duration of disease, female gender, smoking, obesity, other MetS components, prior exposure to other biologics, etc.) did not affect drug persistence in this study. Our results may help rheumatologists to better position IXE in their PsA treatment schemes.

## Figures and Tables

**Table 1:** Main disease characteristics of the study population.

Characteristic	Psoriatic Arthritis n = 72
Age (yrs), mean (SD)	50 (12)
Female, n (%)	52 (72.2)
Disease duration (yrs), median (IQR)	5 (3–9)
Anti-TNFα prior to IXE, n (%)	60 (83.3)
csDMARDs prior to IXE, n (%)	42 (58.3)
tsDMARDs prior to IXE, n (%)	24 (33.3)
Other biologics prior to IXE, n (%)	8 (11.1)
Weight, median (IQR)	77 (65–85)
Obesity (BMI > 30), n (%)	20 (27.8)
Smoker, n (%)	31 (43.1)
Hypertension, n (%)	21 (29.2)
Dyslipidemia, n (%)	25 (34.7)
Diabetes, n (%)	10 (13.9)
COPD, n (%)	2 (2.7)
Cardiovascular disease *	2 (2.7)
Ischemic heart disease	4 (5.5)
Depression	39 (54.2)
Chronic kidney disease	2 (2.7)
Chronic liver disease	1 (1.4)
DAPSA, mean (SD)	9.8 (6.8)
PsAID, mean (SD)	2.7 (2.2)
PASI, mean (SD)	1.8 (0.7)

SD: standard deviation, yrs: years, IQR: interquartile range, TNF: tumor necrosis factor, IXE: ixekizumab, BMI: body mass index, csDMARDS: conventional synthetic disease-modifying anti-rheumatic drugs (methotrexate, leflunomide, sulphasalazine), tsDMARDS: targeted synthetic disease-modifying anti-rheumatic drugs (apremilast, tofacinitib), COPD: chronic obstructive pulmonary disease, DAPSA: disease activity index for psoriatic arthritis, PsAID: psoriatic arthritis impact of disease, PASI: psoriasis area and severity index. * Myocardial infarction and/or cerebrovascular event.

**Table 2:** Main adverse events identified during the study.

Adverse Event	n: 72 (%)	Withdrawal
Gastrointestinal disorders *	5 (6.9)	4/5
General symptoms and local injection site reactions	10 (13.8)	2/10
Disorders of the skin and subcutaneous tissue	5 (6.9)	3/5
Infections **	5 (6.9)	4/5

Cells include n (%). Withdrawal: no. patients in whom the adverse event led to discontinuation of the drug. * Including one case of Crohn’s disease. ** Including candida infections.

## Data Availability

The data on which this study is based are stored in databases. This information is available to third parties on reasonable demand.
